# Knowledge Graphs of Kawasaki Disease

**DOI:** 10.1007/s13755-020-00130-8

**Published:** 2021-02-27

**Authors:** Zhisheng Huang, Qing Hu, Mingqun Liao, Cong Miao, Chengyi Wang, Guanghua Liu

**Affiliations:** 1 Knowledge Representation and Reasoning (KR&R) Group, Vrije Universiteit Amsterdam, Amsterdam, Netherlands; 2 School of Computer Science and Engineering, Wuhan University of Science and Technology, Wuhan, China; 3 Ztone International BV, Purmerend, The Netherlands; 4 Ztone Fujian, Fuzhou City, China; 5 Fujian Provincial Maternity and Children’s Hospital, Affiliated Hospital of Fujian Medical University, Fuzhou, China; 6 Engineering Research Center for Medical Data Mining and Application of Fujian, Fujian, China

**Keywords:** Knowledge graph, Kawasaki Disease, Semantic technology

## Abstract

Kawasaki Disease is a vasculitis syndrome that is extremely harmful to children. Kawasaki Disease can cause severe symptoms of ischemic heart disease or develop into ischemic heart disease, leading to death in children. Researchers and clinicians need to analyze various knowledge and data resources to explore aspects of Kawasaki Disease. Knowledge Graphs have become an important AI approach to integrating various types of complex knowledge and data resources. In this paper, we present an approach for the construction of Knowledge Graphs of Kawasaki Disease. It integrates a wide range of knowledge resources related to Kawasaki Disease, including clinical guidelines, clinical trials, drug knowledge bases, medical literature, and others. It provides a basic integration foundation of knowledge and data concerning Kawasaki Disease for clinical study. In this paper, we will show that this disease-specific Knowledge Graphs are useful for exploring various aspects of Kawasaki Disease.

## Introduction

Kawasaki Disease is one of the high-risk diseases in infants and young children, which causes severe physical and mental damage to children and huge economic burden on the family and society. The biggest harm of Kawasaki Disease is the damage to coronary arteries, which is the main cause of coronary artery disease in children and also in adulthood. However, it is extremely difficult to predict and timely monitor the high-risk factors of Kawasaki Disease. After being diagnosed with Kawasaki Disease, patients often miss the optimal period for the treatment. Clinicians need to analyze various complex knowledge and data resources related to Kawasaki Disease to obtain information to make effective clinical decisions.

The term “Knowledge Graph” is widely used to refer to a large scale semantic network consisting of entities and concepts as well as the semantic relationships among them, using representation languages such as RDF and RDF Schema [1]. Such knowledge graphs are used in the construction of many knowledge-based applications in medicine, such as extracting information from patient records [2], personalised medicine [3], support for co-morbidity analysis [4], data integration on drugs and their interactions [5], and many others [6–9].

Medical knowledge graphs typically cover very wide areas of medical knowledge: all proteins (UniProt), many known disease–gene associations (DisGeNet), as many drugs as possible (Drugbank), as many drug–drug interactions as are known (Sider), and massively integrated knowledge graphs such as Bio2RDF^1^ and Linked Life Data.^2^ We have constructed Knowledge Graphs of Kawasaki Disease, or alternatively called Kawasaki Disease Knowledge Graphs (KDKG). The KDKG integrates a variety of knowledge resources related to Kawasaki Disease, including clinical guidelines, clinical trials, medical literature, drug knowledge base, and clinical ontology knowledge bases such as SNOMED CT^3^ in clinical medical concept terminology, etc. By constructing Knowledge Graphs of Kawasaki Disease, comprehensive knowledge can be effectively transformed into well-structured knowledge. It enables us to adopt a knowledge base method and obtain corresponding knowledge quickly and accurately through semantic queries. It provides well-structured data infrastructure for clinical decision support.

In this paper, we describe how Knowledge Graphs of Kawasaki Disease can be constructed, investigate how we can use Knowledge Graphs of Kawasaki Disease to explore various aspects of Kawasaki Disease, and discuss their medical potentials for clinical decision support of the disease. The main contributions of this paper are: (i) we show how to construct Knowledge Graphs of Kawasaki Disease, (ii) we present that Knowledge Graphs can provide a more efficient way on the literature retrieval for the study of Kawasaki Disease, (iii) we show that Knowledge Graphs can be used to explore various aspects of Kawasaki Disease.

The rest of paper is organized as follows: First We illustrate the general ideas of Knowledge Graphs in “Knowledge graphs ” section. In “Knowledge graphs of Kawasaki Disease” section, we introduce the general architecture of Knowledge Graphs of Kawasaki Disease to show how to integrate various knowledge/data resources about Kawasaki Disease (e.g., clinical trials, medical publications, clinical guidelines, etc.). KDKG provides a data infrastructure to explore the relationship among various knowledge and data-sources about Kawasaki Disease. In “Knowledge graphs for semantic search” section we discuss how to use Knowledge Graphs of Kawasaki Disease for semantic search. “Discussion and Conclusion” section discusses the future work and concludes the paper.

## Knowledge graphs

Following commonly used technology, we will construct our knowledge graph as an RDF graph. Formally, an RDF graph is a collection of triples $\langle s,p,o \rangle,$ each consisting of a subject *s*, a predicate *p* and an object *o*. Each triple represents a statement of a relationship *p* between the things denoted by the nodes *s* and *o* that it links. Identifiers for both *p*, *s* and *o* are URI’s (Uniform Resource Identifier), allowing triples in one knowledge graph to refer to elements in another knowledge graph that resides in a physically different location. Besides a URI, the object *o* of a triple $\langle s,p,o \rangle$ can also be a literal (roughly: a string or any other XML-sanctioned datatype). Whereas objects that are denoted by URI’s can themselves be the subject of other triples (giving rise to the graph construction), literals cannot be themselves the subject of other triples. Summarising, let *U* be the set of all URI, and *L* be the set of all literals. A knowledge graph *K* can be defined as a set of three-place tuples $\langle s,p,o \rangle,$ with $s,p \in U$ and $o \in U \cup L.$

The languages RDF and RDF Schema [1] assign a fixed semantics to some of the predicates *p*. Examples of these are the predicates rdf:type to denote membership of a type, rdfs:subClassOf to denote (transitive) containment of subclasses, rdfs:domain and rdfs:range to denote membership of any subject resp. object of a given predicate to a specified type. For richer semantic relations, knowledge graphs can contain language constructions from the Web Ontology Language OWL [10], which allow the enforcement of disjointness between classes, cardinality constraints on relations and others. These (and other) predefined predicates allow for automatic inference of additional triples from a given knowledge graph. For a full specification of the syntax and semantics of knowledge graphs in RDF, RDF Schema and OWL we refer to the specification documents of these languages as cited above.

As a simple example, we state the basic properties of a clinical trial in Kawasaki Disease:
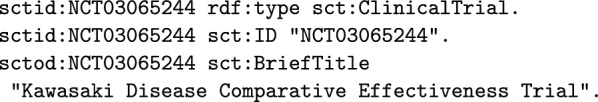


This example shows the use of name space abbreviations, writing rdf:type instead of http://www.w3.org/1999/02/22-rdf-syntax-ns#type. A triple can be represented by using a prefix for a name space abbreviation. For example, we can use the prefix “rdf” to denote the string http://www.w3.org/1999/02/22-rdf-syntax-ns#, “sctid” to denote the string http://wasp.cs.vu.nl/sct/id#, and “sct” to denote the string http://wasp.cs.vu.nl/sct/sct#. The first triple in the example above can be written as

$$\langle sctid:NCT03065244, rdf:type, sct:ClinicalTrial\rangle$$

These triples state that that sctid:NCT03065244 is an instance of the concept ClinicalTrial with the given name (second triple) and title (third triple).

**Fig. 1:**
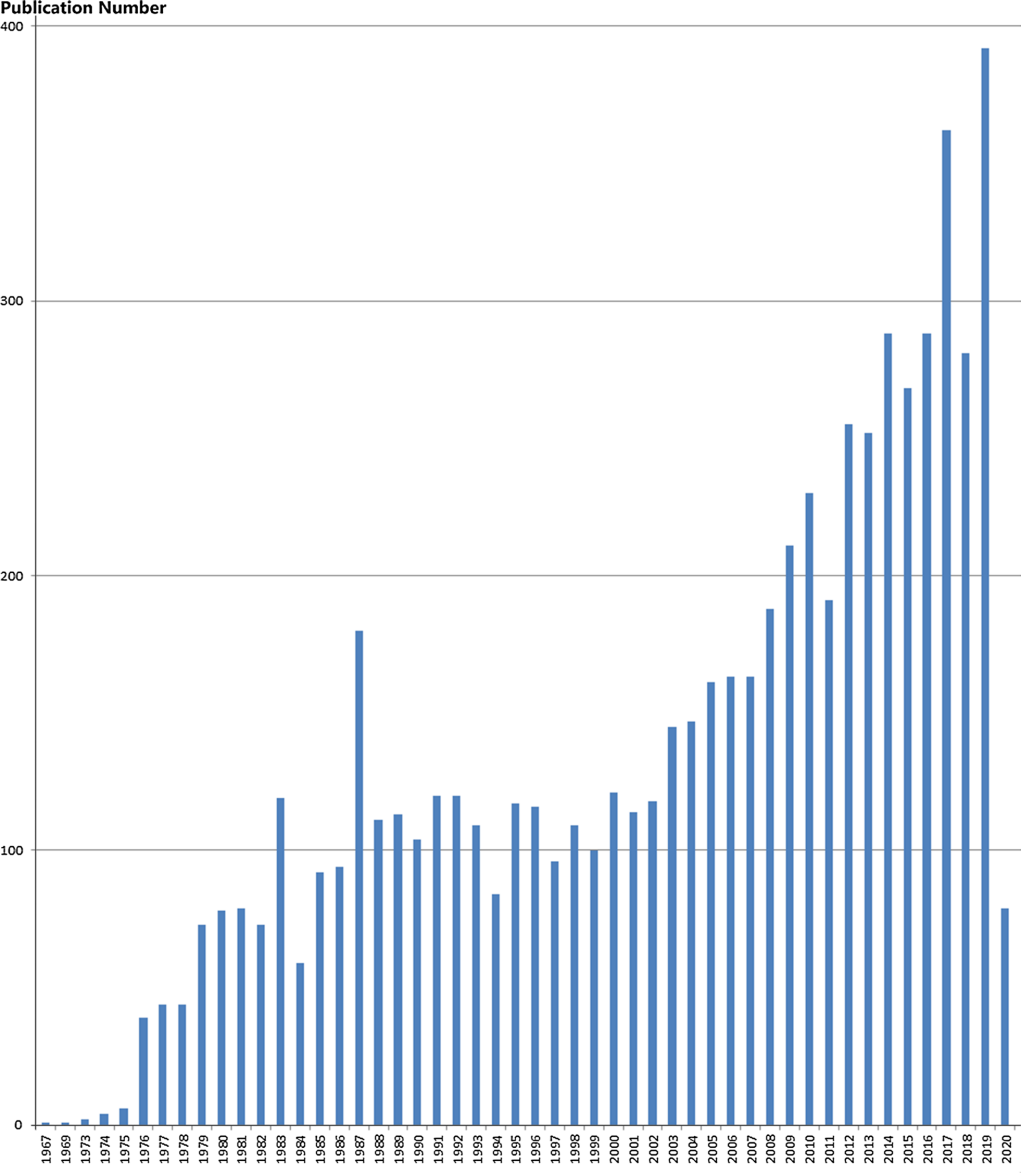
Papers about Kawasaki Disease by years

## Knowledge graphs of Kawasaki Disease

In the present version (version 0.5) of Knowledge Graphs of Kawasaki Disease [11], we focus on the following knowledge/data resources:

- *PubMed* We used the keyword "Kawasaki Disease" to search for publications in PubMed^4^ obtained 6440 publications. This PubMed data set contains the basic data of a publication: authors, title of paper, journal name, publication date, abstract of the paper, PubMed ID (PMID), DOI, and its MeSH Terms. We converted the XML meta-data of PubMed which are downloaded from the PubMed website into RDF Ntriples Data. Figure 1 shows the year distribution of the papers about Kawasaki Disease. From the figure we can see that the publications on Kawasaki Disease research are increasing significantly in recent years.
- *Clinical Trials*. We searched for clinical trials on Kawasaki Disease on ClinicalTrials.gov^5^ and downloaded 38 studies with all study and result fields as XML data. We then converted those XML data into RDF triples. The basic data of clinical trials includes Trial ID, title of trial, agency, authority, brief summary, detailed description, completion status, starting date, study type and design, phase, eligibility criteria, reference, MeSH terms.
- *Semantic Annotations of the publications*. Abstracts of papers in Kawasaki Disease medical literature are expressed as free text. In order for our system to effectively and accurately analyze the corresponding medical literature, we need to perform semantic annotations on the abstracts of these papers. We use XMedlan [12], a semantic annotation tool medical literature, to make the semantic annotations of free text with the UMLS concepts (CUI) and the clinical terminologies SNOMED CT, and generate the corresponding semantic data. One of the benefits of using ontology-based semantic annotation is that the corresponding ontology already provides all synonymous conceptual labels of medical concepts. For example, for the concept “Kawasaki Disease”, the corresponding synonymous conceptual labels include: acute febrile skin mucosal lymph node syndrome (Acute febrile mucocutaneous lymph node syndrome), Kawasaki’s syndrome, Kawasakis mucocutaneous lymph node syndrome, and various abbreviations of the concept, such as MCLS, MLNS, KD and other forms. As long as we use one of the concept labels, the system can identify all other concept labels and their abbreviations, so as to avoid the problem of finding all relevant documents through multiple queries.
- *Medical Guidelines* We have obtained several one guidelines of Kawasaki Disease. So far we have converted only the text of the American guidelines of Kawasaki Disease (2017) into the semantic representation which are based our design for evidence-based medical guidelines [13]. The basic data about an evidence-based guideline includes title, publication year, version, topics, conclusion evidence class, and its evidences (as PubMed IDs).
- *DrugBank* The DrugBank database^6^ is a bio-informatics and chem-informatics resource that combines detailed drug data with comprehensive drug target (i.e. sequence, structure, and pathway) information. The current version of the database contains 4770 drugs.
- *SIDER* SIDER contains information on marketed drugs and their recorded side-effects^7^ The available information includes side effect frequency, drug and side effect classifications as well as links to further information, for example drug-target relations.
- *SNOMED CT*. SNOMED CT provides the clinical terminology and the concept hierarchy statements.

The current version of Knowledge Graphs of Kawasaki Disease (version 0.50) consists of the RDF representation of the above knowledge resources. A summary of Knowledge Graphs of Kawasaki Disease is shown in Table 1. This shows that the resulting knowledge graph is only of moderate size (10,146,311 triples), whereas many of the original knowledge graphs are many times larger than this (30 M–100 M triples).

**Table 1:** Kawasaki Disease KG version 0.5

Knowledge resource	Number of data item	Number of triple
Clinical Trial	31 trials	4299
PubMed on Kawasaki Disease	6440 papers	276,066
Semantic annotation on Kawasaki Disease	6440 papers	385,9341
Medical guidelines	1 guideline	1211
Drug Bank	4770 drugs	766,920
SIDER	1169 drugs	193,249
SNOMED CT		5,045,225
Total		10,146,311

We use the following four methods to integrate the various knowledge resources.

- *Direct Entity identification*. Some knowledge resources refer to the same entity with identical names, e.g. the PubMed IDs used in both PubMed and the clinical trials. Such identical entities are obvious links between these knowledge sources.
- *Direct Concept identification*. Numerous knowledge resources can be integrated by using direct concept identification. For example, both a publication in PubMed and a clinical trial are annotated with SNOMED CT and UMLS terms. This provides us with a way to detect a relationship between a clinical trial and a publication directly.
- *Semantic Annotation with an NLP tool*. We used Xerox’s NLP tool XMedlan [12, 14] for semantically annotating medical text (both concept identification and relation extraction) with medical terminologies such as SNOMED CT. We use XMedLan rather than similar terminology-based concept identifiers, such as the MetaMap system [15] or the BioPortal text annotator,^8^ because it is easier to adapt. XMedlan can be customized using a single command line with any subset from UMLS-integrated terminologies and even with in-house, non-standard terminologies [12]. Furthermore, although XMedLan and MetaMap seem to have comparable concept identification capabilities on the ShARe/CLEF-2013 corpus (see [14]), we have never had any computational efficiency issues with XMedLan, while MetaMap can have such issues [15, 16]. By using Xerox’s NLP tool, we can obtain the semantic annotations and extracted relations over free medical text in those knowledge resources.
- *Semantic Queries with regular expressions*. The previous three approaches are off-line approaches to integrate knowledge sources. Semantic Queries with regular expressions are an online approach, because such queries find relationships among knowledge resources at query time. Although online method lead to more latency when getting query results, they do provide a method to detect a connection among different knowledge resources based on free text.

**Fig. 2:**
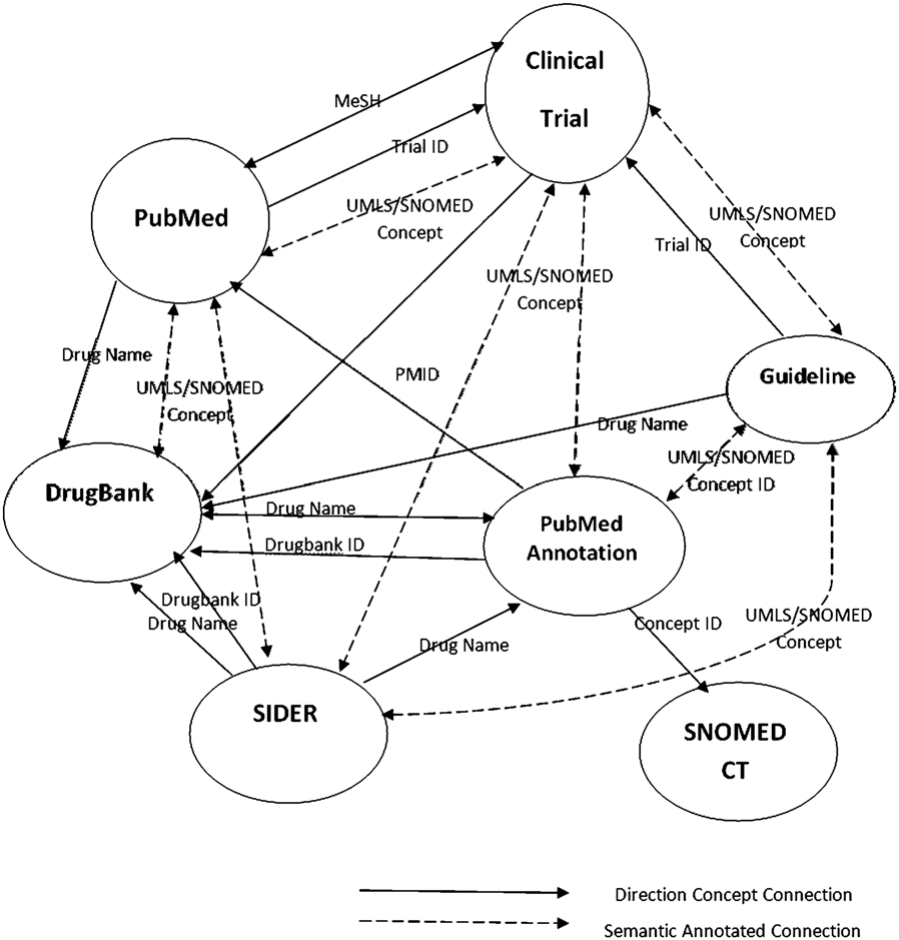
Integration of knowledge graphs of Kawasaki Disease

Figure 2 shows the connectivity of the Knowledge Graphs via direct concept identification and semantic annotation with medical concepts. An arrow denotes a direct concept connection from one knowledge resource to another via a property, and a dashed arrow denotes a semantic annotation connection via a concept identification in a medical terminology by using an NLP tool. The figure shows that our set of knowledge resources is well integrated.

## Knowledge graphs for semantic search

In this section, we will discuss how Knowledge Graphs of Kawasaki Disease can provide more efficient ways for the semantic search in the study of Kawasaki Disease. We will illustrate the ideas by showing several SPARQL queries for the semantic research. However, we would not expect medical professionals such as doctors/researchers who can design SPARQL queries to explore the knowledge graphs. Making those queries requires knowledge of semantic web standards and the structure of the knowledge graph, and we would not expect a doctor/researcher to formulate those queries. Instead, such queries can be designed by the system developers and then used as a template for a user to change the parameters in the template to make their own queries. Furthermore, the queries can be wrapped in a user-friendly GUI.

We have implemented the system of Knowledge Graphs of Kawasaki Disease, which is built on the semantic processing platform GraphDB.^9^ It integrates all kinds knowledge and data resources of Kawasaki Disease as described above, and can provide corresponding semantic queries. It serves as the basis of a knowledge management and service platform system for Kawasaki Disease. It is expected to provide the infrastructure for research, diagnosis, consultation, and clinical decision support for Kawasaki Disease.

Aiming at the knowledge graph system, we can design different semantic query modes and provide semantic queries involving different knowledge sources. Here are some use cases of Knowledge Graphs of Kawasaki Disease.

- *Case 1* We want to find a clinical trial of Kawasaki Disease, which uses a drug that targets the calcium-dependent phospholipid binding target. Such a query must first determine which drugs are targeted at the target from the drug knowledge base, and then discover which drugs are being tested as interventions from the clinical trial knowledge base. Such a multiple query can be obtained directly through a SPARQL semantic query:
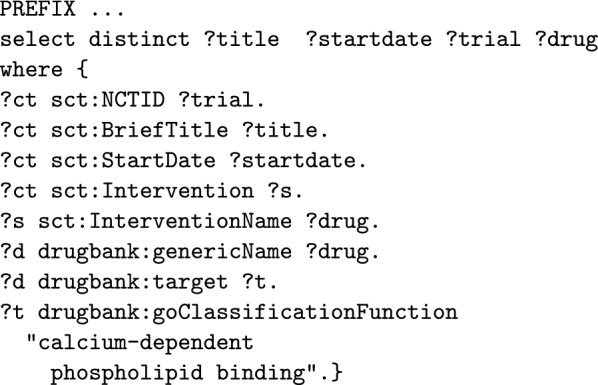
  From such a semantic query, we can find the clinical trial NCT03200561 (A Trial to Evaluate the Efficacy of Immunoglobulin Plus Steroid for Prevention of Coronary Artery Abnormalities in Taiwanese Refractory Kawasaki Disease (RAST Study)), which started on October 17, 2013. The drug used for the intervention is Prednisolone.
- *Case 2* To address the clinical question: whether coronary vascular smooth muscle cells or endothelial cells proliferate or undergo apoptosis after coronary artery injury of Kawasaki Disease. We can write part of this query as the following query: Is there a large number of vascular smooth muscle apoptosis after Kawasaki Disease coronary injury? Its corresponding simplified English expression is Apoptosis of vascular smooth muscle more? The tool we implemented can automatically translate such a query expressed in English natural language into the following SPARQL query (ie, semantic query) to find and discuss Kawasaki Disease and cells Papers on the relationship between apoptosis:
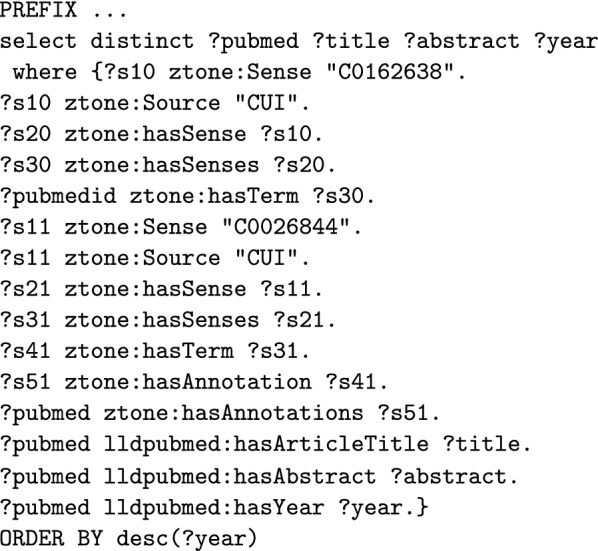
  Our knowledge graph system is able to find two papers, both of which were published in 2017. Through the above semantic query, we can know that the main relationship between Kawasaki Disease and cell decay.
- *Case 3* The international medical community has never found the true cause of Kawasaki Disease. A natural idea is whether Kawasaki Disease is associated with a certain bacteria or virus. Through semantic search, we can quickly and easily discover which viruses or bacteria are often discussed with Kawasaki Disease over the years. The following SPARQL is a semantic query for the association between viruses and Kawasaki Disease:
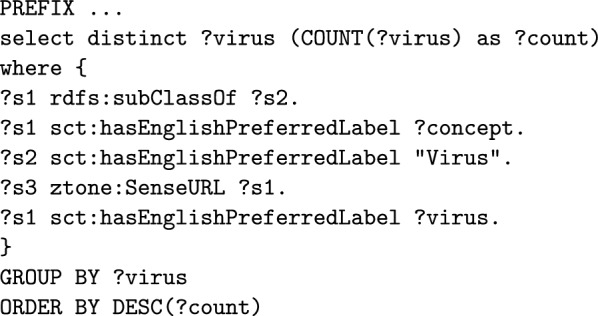
  The returned results of the query above are shown in Table 2. The results tell us that Herpes zoster is the most frequently mentioned virus associated with Kawasaki Disease (175 times), followed by Retroviridae, mentioned 168 times. It is worth noting that the above semantic query needs to traverse all the conceptual structure levels of virus in the clinical concept term set SNOMED CT to obtain complete results. This is obtained through the inference analysis of *rdfs* : *subClassOf* by the knowledge graph inference engine embedded in the semantic platform, which also fully shows the value of medical knowledge graph.

**Table 2:** Suspected virus of Kawasaki Disease

?virus	?count
Herpes zoster	175
Retroviridae	168
RNA tumour virus	168
Respiratory syncytial virus	162
Herpesviridae	78
Parvovirus	63
Influenzavirus	63
Parvovirus	63
Human coronavirus	40
...	...
- *Case 4* Immunoglobulin (IVIG) is one of the main treatment methods for Kawasaki Disease. However, the prevalence of immunoglobulin resistance in the children with Kawasaki Disease was 30%. We are interested in the exploration of various aspects of immunoglobulin resistance in Kawasaki Disease. First of all, we want to search for all the papers which are relevant with IVIG resistance in Kawasaki Disease. However, we cannot find any concept which corresponds with "IVIG resistance" in UMLS and SNOMED CT terminologies. Fortunately we find the MeSH heading "Drug Resistance" in the meta-data of PubMed, which have been covered by the semantic data of the publications in the knowledge graphs. Thus, we can use the following SPARQL query with UMLS ID "C0085297" for the concept "IVIG" in the search:
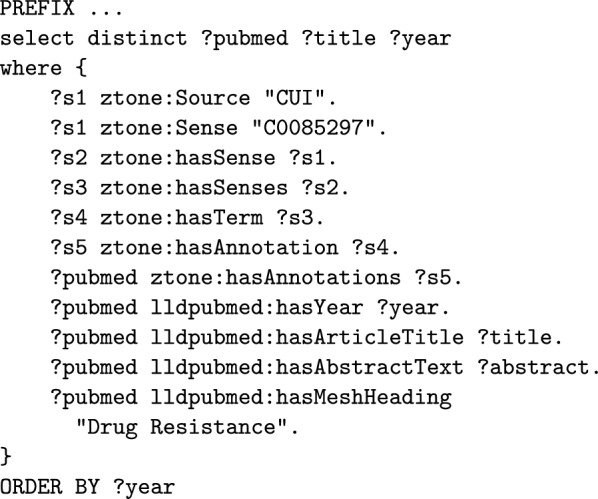
 which can returns 78 papers in that semantic search.

## Discussion and conclusion

We have introduced a method for constructing a Knowledge Graphs of Kawasaki Disease. Through the knowlege/data integration technology based on semantic technology, we can integrate various knowledge/data resources of Kawasaki Disease that have been loosely connected into a well-structured ones, thereby providing a data infrastructure for exploring various aspects of Kawasaki Disease. We have shown several cases how Knowledge Graphs of Kawasaki Disease can be used for efficient semantic queries in Kawasaki Disease.

In future work, we will conduct more comprehensive meta-analysis on various aspects of Kawasaki Disease by using Knowledge Graphs of Kawasaki Disease. The interesting meta-analysis includes comprehensive discussion on bio-markers and their clinical implications for Immunoglobulin Resistance and analysis on the possibilities to find the pathogens and poisons which may lead to Kawasaki Disease.

## Footnotes

1 http://bio2rdf.org/.

2 http://linkedlifedata.com/.

3 http://www.snomed.org/.

4 https://www.ncbi.nlm.nih.gov/pubmed/?term=KawasakiDisease.

5 https://clinicaltrials.gov.

6 https://www.drugbank.ca/.

7 http://sideeffects.embl.de/.

8 https://bioportal.bioontology.org/annotator.

9 https://www.ontotext.com/products/graphdb/.
